# Telmisartan protects against microvascular dysfunction during myocardial ischemia/reperfusion injury by activation of peroxisome proliferator-activated receptor gamma

**DOI:** 10.1186/1471-2261-13-39

**Published:** 2013-06-05

**Authors:** Xiao-Cong Zeng, Xing-San Li, Hong Wen

**Affiliations:** 1 Department of Cardiology, the First Affiliated Hospital of Guangxi Medical University, Nanning, Guangxi, 530021, People’s Republic of China

**Keywords:** Ischemia/reperfusion, Microvascular dysfunction, Telmisartan, Peroxisome proliferator-activated receptor gamma

## Abstract

**Background:**

We investigated the potential of telmisartan to improve microvascular dysfunction induced by myocardial ischemia/reperfusion (I/R) injury by activating the peroxisome proliferator-activated receptor gamma (PPARG) pathway.

**Methods:**

Forty-eight male rabbits were randomly allocated into sham-operated, I/R, GW9662, telmisartan, telmisartan–GW9662, or candesartan groups. Rabbits were anesthetized, and the left anterior descending coronary artery (LAD) was ligated for 60 minutes. Following reperfusion for 6 hours, angiotensin II content of the heart was determined using radioimmunoassay. Myocardial neutrophil accumulation and microvessel cross-sectional area were examined histologically. Myocardial capillaries were examined with transmission electron microscopy. Intercellular adhesion molecule-1 (ICAM-1) and vascular cell adhesion molecule-1 (VCAM-1) in the myocardium were measured using enzyme-linked immunosorbent assay. Western blot was utilized for investigating the expression of nuclear factor kappa-light-chain-enhancer of activated B cells (NF-***κ***B) and PPARG.

**Results:**

Angiotensin II concentration was significantly increased in all treatment groups compared with the sham-operated group (*P* < 0.05, all). Accumulation of polymorphonuclear neutrophils was significantly lower, while microvessel cross-sectional area was significantly higher in the telmisartan, telmisartan-GW9662, and candesartan groups compared with the I/R group (*P* < 0.05). ICAM-1 and VCAM-1 levels were also significantly lower, and correlated with lower NF-***κ***B expression in these groups. The effects were the most significant in the telmisartan group compared with the telmisartan–GW9662 and candesartan groups. Telmisartan significantly increased PPARG protein expression compared with all other groups (*P* < 0.05, all).

**Conclusions:**

Except for the typical effects of angiotensin II-receptor blocker, telmisartan improved microvascular dysfunction during myocardial I/R injury via the PPARG pathway.

## Background

Reperfusion strategies, including primary percutaneous coronary interventions and thrombolytic therapy, are associated with a significant decrease in both short- and long-term mortality of patients with acute ST segment-elevated myocardial infarction [1,2]. Recanalization of an infarct-related artery or percutaneous coronary intervention however does not necessarily guarantee adequate myocardial perfusion, despite angiographic evidence that the target stenosis is adequately removed or bypassed [3]. This phenomenon is termed “no-reflow” and results in a higher incidence of adverse clinical outcomes, including increased left ventricular remodeling, malignant ventricular arrhythmias, heart failure, and cardiac death [4]. Although the mechanisms responsible for no-reflow have not been completely delineated, those that are known to underlie myocardial hypoperfusion have been proposed, including capillary plugging by neutrophils, coronary spasm, intracellular or interstitial edema, microvascular flow disturbances due to platelet aggregation, distal embolization of arteriosclerotic debris, and oxygen free radical–mediated endothelial injury [5-7]. Clinical studies have suggested that microvascular dysfunction is indicative of no-reflow [8].

Although no-reflow clearly results in a poor prognosis, little guidance is available for the treatment of this phenomenon. An ideal treatment strategy would involve drugs with multiple actions on microvascular damage induced by ischemia/reperfusion (I/R) [9]. Akiyoshi et al. [10] showed that an angiotensin type 1 receptor (AGTR1) blockade prevents microvascular dysfunction induced by I/R injury. Other recent studies have suggested that peroxisome proliferator-activated receptor gamma (PPARG) agonists have a protective role subsequent to I/R damage, and the cardioprotective effects of PPARG activation are most likely due to inhibition of the inflammatory response [11,12].

Benson et al. [13] reported that the highly lipophilic angiotensin receptor blocker (ARB) telmisartan can influence PPARG target gene expression by functioning as a partial agonist of PPARG. Telmisartan also induced PPARG activity in the absence of AGTR1, demonstrating that its activation is independent of an AGTR1 blockade [14]. Base on the theory, telmisartan may have more effects than other ARB without PPARG activity, such as candesartan. Ikejima et al. [15] reported that telmisartan has additional effects on nitric oxide bioavailability and atherosclerotic changes via the PPARG pathway in genetically hyperlipidemic rabbits. It has also been reported that telmisartan reduced oxidative stress, apoptosis and improved cardiac function through PPARG-mediated effects in diabetic rats with myocardial infarction [16]. Telmisartan has shown new pleiotropic actions through induction of PPARG activity, providing a potential treatment mechanism for microvascular impedance induced by I/R injury.

The present study investigated the role of telmisartan in preventing microvascular dysfunction, in addition to its role as an ARB, through its PPARG-mediated effects during a rabbit myocardial I/R injury model results from a protocol of 60 minutes of left anterior descending coronary artery (LAD) ischemia followed by 6 hours of reperfusion.

## Methods

### Experimental animals

Forty-eight healthy adult male New Zealand white rabbits (2.0–2.5 kg) were purchased from the Center for Experimental Animals, Guangxi Medical University, Nanning City, China. Animals were housed at 25 ± 2°C and 60 ± 5% humidity, and exposed to a 12:12 h light–dark cycle with pellet food and tap water *ad libitum*. Animal experiments were performed in accordance with the European Community Guidelines for the Care and Use of Animals, and the Institutional Ethics Committee for Animal Usage approved the research protocol.

### Modeling and grouping

The 48 rabbits were randomly apportioned into six groups (n = 8, each). Rabbits of the 6 experimental groups were given daily doses of the following by gastric gavage for 14 days: The sham-operated and I/R groups were administered the vehicle only (0.5% carboxymethyl cellulose sodium and 10% dimethyl sulfoxide solution). The GW9662 group received the PPARG antagonist GW9662 (0.5 mg · kg^-1^ · d^-1^; Sigma, St. Louis, MO, USA). The telmisartan group received the angiotensin receptor blocker telmisartan (5 mg · kg^-1^ · d^-1^; Micardis®, Boehringer Ingelheim). The telmisartan–GW9662 group was given telmisartan (5 mg · kg^-1^ · d^-1^) and GW9662 (0.5 mg · kg^-1^ · d^-1^). The candesartan group received candesartan (5 mg · kg^-1^ · d^-1^; Atacand, AstraZeneca), an angiotensin receptor blocker without PPARG activity [15].

All animals were anesthetized using 20% urethane via an ear marginal vein injection. Following midline thoracotomy, a 4–0 silk ligature was placed under the LAD. The ends of the suture were threaded through polyethylene tubing to form a snare. The ends of the suture were pulled tight and a hemostat was used to clamp the snare to occlude the coronary artery. After 60 min of ischemia, the ligature was untied and the snare was loosened, allowing the ischemic myocardium to reperfuse for 6 h [17]. Sham-operated animals were subjected to all of the previously described procedures except ligation of the LAD.

### Tissue sampling

A cardiectomy was performed, the atrial appendage, atrium cordis and right ventricle were removed, and the left ventricle was cut into three transverse sections from the apex to the base. The base portion of the left ventricle was cut into a sample of approximately 1 mm^3^ subendocardial tissue for examination of the capillary ultrastructure by electron microscopy. The section from the mid-portion was used for morphological examination by Hematoxylin and eosin (H&E) and immunohistochemistry staining. The apical portion was frozen in liquid nitrogen and stored at −80°C for assay of myocardial angiotensin-II concentrations using radioimmunoassay, for determination of myocardial intercellular adhesion molecule-1 (ICAM-1) and vascular cell adhesion molecule-1 (VCAM-1) concentrations by enzyme-linked immunosorbant assay (ELISA), for determination of nuclear factor kappa-light-chain-enhancer of activated B cells (NF-***κ***B) and PPARG expression by western immunoblot analyses.

### Myocardial angiotensin-II concentration

Myocardial angiotensin-II concentrations were measured via an iodine-125 radioimmunoassay using an angiotensin-II radioimmunoassay kit (Beijing North Institute of Biological Technology, Beijing, China) in accordance with the manufacturer’s instructions [18]. Radioactivity was measured with a GC-1200***γ*** radioimmunoassay counter (Anhui USTC Zonkia Scientific Instruments, China). Protein concentrations were determined with the Lowry method [19].

### Morphological examination and assessment of myocardial neutrophil accumulation

Myocardial morphological and neutrophil accumulation was examined using H&E staining. A portion of myocardial tissue from the I/R zone (i.e., the left ventricle anterior wall) was removed, fixed in 10% formalin, and embedded in paraffin in accordance with standard laboratory procedures. After cutting the sample into thin slices, it was stained with H&E. The number of polymorphonuclear neutrophils (PMNs) infiltrating into the myocardial tissue, and the number of PMNs adhering to the capillaries, were determined per high-power field [20]. For each heart, PMNs were counted in six fields using three independent tissue sections by an observer blinded to the experimental treatment. Counts were averaged.

### Microvessel cross-sectional area

Myocardial microvessels were highlighted using anti- Cluster of differentiation (CD)34 (mouse anti-human immunoglobulin antibody, Maixin Biology, Fuzhou, China) immunostaining. A portion of myocardial tissue from the I/R zone was removed, fixed in 10% formalin, and embedded in paraffin in accordance with standard laboratory procedures. After cutting the sample into thin slices, it was stained using the streptavidin-peroxidase (Maixin Biology, Fuzhou, China) immunohistochemical method [21]. A pathological image analysis system (DMR + Q550, Leica, Wetzlar, Germany) was used to calculate the cross-sectional area of the microvessels (***μ***m^2^), at 400× magnification from 5 visual fields with the highest density of microvessels [22]. Microvessel cross-sectional area was considered the mean obtained in these five fields.

### Capillary ultrastructure

The ultrastructure of the capillaries was examined using transmission electron microscopy (H-500, Hitachi, Tokyo, Japan) of subendocardial tissue from ischemic regions of the hearts that were subjected to the I/R protocol, as described above. The tissues were prepared for transmission electron microscopy as described previously [23].

### Myocardial ICAM-1 and VCAM-1 concentrations

Myocardial ICAM-1 and VCAM-1 concentrations were determined using ELISA. Frozen left ventricle tissue samples were homogenized in lysis buffer containing Tris–HCl 50 mM, ethylenediaminetetraacetic acid 1 mM, and 5% sucrose (pH 7.4). The protein concentration was determined using the Lowry method [19]. ICAM-1 and VCAM-1 were determined using commercially available ELISA kits (RapidBio Lab, Calabasas, California, USA) [24].

### Myocardial NF-***κ***B and PPARG protein expression

The NF-***κ***B and PPARG protein expression were examined using western blot analysis. Proteins were resolved by sodium dodecyl sulfate-polyacrylamide gel electrophoresis, transferred onto a nitrocellulose membrane, and incubated serially with the rabbit anti-human NF-***κ***B, rabbit anti-human PPARG, and rabbit anti-human actin polyclonal antibodies (Santa Cruz Biotechnology, CA, USA) at 4°C overnight. Proteins were detected using horseradish peroxidase-conjugated secondary antibody (Santa Cruz Biotechnology, CA, USA) and visualized using the enhanced chemiluminescence method [25]. The Bio-Rad Gel Doc 2000 imaging system and software were used to calculate the integrated absorbance (IA) of identified bands: IA = Area × Average density [26]. Following normalization to ***β***-actin (ACTB) levels, the ratios of the IAs of NF-***κ***B or PPARG to the Ia of ACTB were used to represent relative levels of activated NF-***κ***B and PPARG.

### Statistical analyses

Data are expressed as mean ± standard deviation. Statistical differences were determined using analysis of variance (ANOVA); *P* < 0.05 was considered statistically significant. All the analyses were performed using SPSS software (version 16; SPSS, Chicago, IL, USA).

## Results

### Animal groups

No statistically significant differences were found in body weights or heart rates among the 6 experimental groups (data not shown).

### Measurement of Angiotensin-II concentration in the left ventricle by Radioimmunoassay

The myocardial angiotensin-II concentrations in the treatment groups were: sham-operated, 152 ± 20 pg/mg; I/R, 221 ± 40 pg/mg; GW9662, 226 ± 19 pg/mg; telmisartan, 235 ± 33 pg/mg; telmisartan–GW9662 227 ± 26 pg/mg; and candesartan 215 ± 39 pg/mg. The angiotensin-II concentrations of all the treatment groups were significantly higher than that of the sham-operated group (*P* < 0.05).

### Assessment of PMN infiltration into the myocardium by H&E staining

In the sham-operated group, the results of H&E staining show that muscle fibers were integrated and nucleoli were normal. The muscle fibers of the I/R and GW9662 groups had extensive edema, necrosis, and breakage, and a large number of dissolved myocyte nucleoli. Also, the number of PMNs infiltrating the area of the reperfused myocardium was increased in the I/R (78.38 ± 10.68/high-power field) and GW9662 (85.38 ± 11.17/high-power field) groups, compared with the sham-operated (2.13 ± 1.81/high-power field).

**Figure 1:**
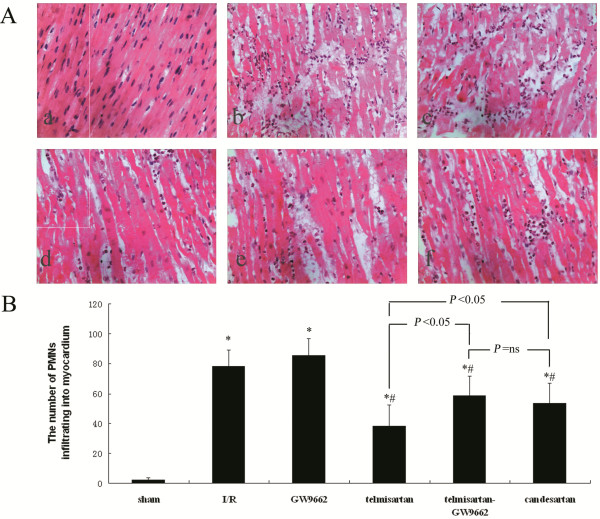
**Assessment of PMN infiltration into the myocardium by H&E staining. A**) Micrographs show PMNs infiltrating into the myocardium: (**a**) sham-operated group; (**b**) I/R group; (**c**) GW9662 group; (**d**) telmisartan group; (**e**) telmisartan–GW9662 group; (**f**) candesartan group; (H&E stain; 400×). **B**) Quantitative analyses of the number of PMNs infiltrating into the myocardium. *P < 0.05 compared with sham-operated; ^#^*P* < 0.05 compared with I/R.

In the telmisartan, telmisartan–GW9662, and candesartan groups, the muscle fibers showed less breakage and edema, along with fewer ruptured nucleoli. There was also significantly fewer PMN infiltration in the myocardium of the telmisartan (38.25 ± 14.11/high-power field), telmisartan–GW9662 (58.63 ± 12.68/high-power field), and candesartan (53.38 ± 13.57/high-power field) groups, compared with the I/R and GW9662 groups. Furthermore, there were significantly fewer PMNs infiltrating the myocardium in the telmisartan group compared with the telmisartan–GW9662 and candesartan groups (Figure 1).

### Assessment of PMN adherence to microvessels by H&E staining

**Figure 2:**
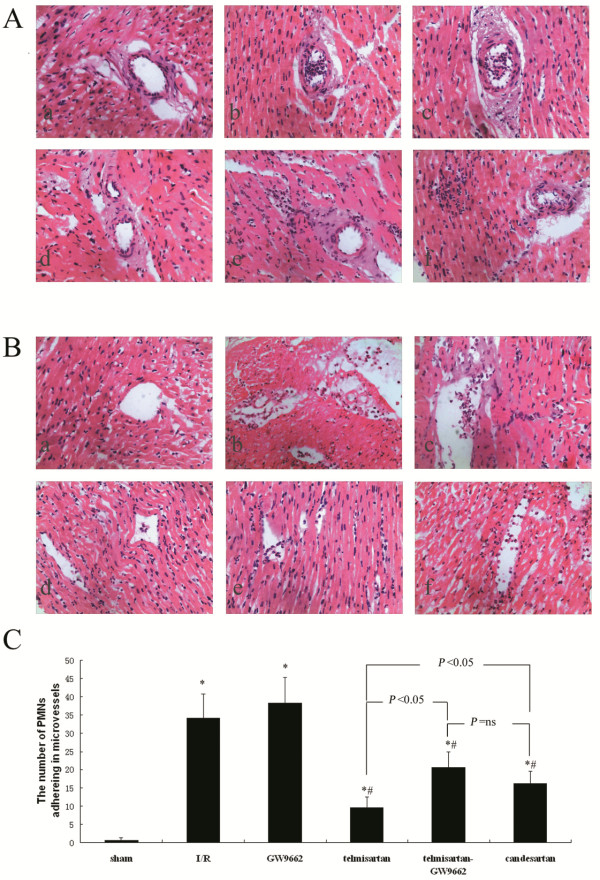
**Assessment of PMN adherence to microvessels by H&E staining. A**) Micrographs showing PMNs adhering to arterioles: (**a**) sham-operated group; (**b**) I/R group; (**c**) GW9662 group; (**d**) telmisartan group; (**e**) telmisartan–GW9662 group; (**f**) candesartan group; (H&E stain; 400×). **B**) Micrographs show PMNs adhering to venules: (**a**) sham-operated group; (**b**) I/R group; (**c**) GW9662 group; (**d**) telmisartan group; (**e**) telmisartan–GW9662 group; (**f**) candesartan group; (H&E stain; 400×). **C**) Quantitative analyses of the number of PMNs adhering to microvessels. *P < 0.05 compared with sham-operated; ^#^*P* < 0.05 compared with I/R.

The results of H&E staining show that the I/R and GW9662 groups had neutrophils primarily adhering and plugging the venules, while occasionally also affecting the arterioles. The number of PMNs adhering to the microvessels were also increased in the I/R (34.25 ± 6.41/high-power field) and GW9662 (38.25 ± 6.98/high-power field) groups. However, in the telmisartan (9.50 ± 3.02/high-power field), telmisartan–GW9662 (20.63 ± 4.27/high-power field), and candesartan (16.25 ± 3.37/high-power field) groups, the neutrophils were observed adhering and plugging the venules, but scarcely present in the arterioles, and there were significantly fewer PMNs adhering to the microvessels in these groups compared with the I/R and GW9662 groups. The telmisartan group had significantly fewer PMNs adhering to the microvessels compared with either the telmisartan–GW9662 or candesartan group (Figure 2).

### CD34-immunostaining of microvessels and microvessel cross-sectional area

**Figure 3:**
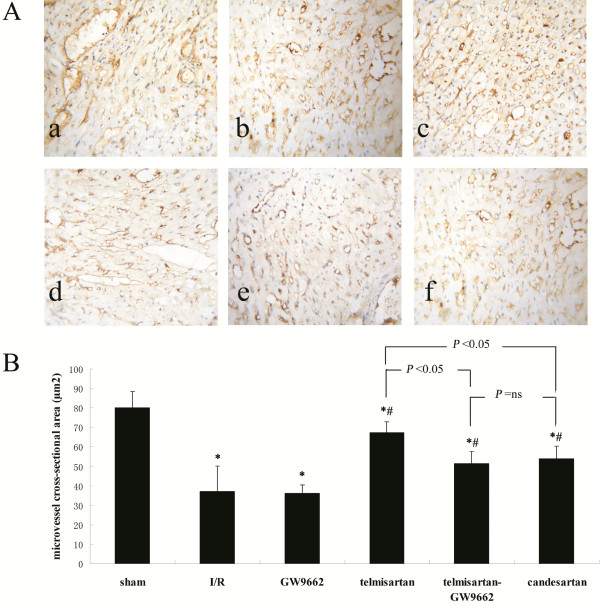
**Assessment of microvessel cross-sectional area by CD34-immunostaining. A**) Photomicrographs of myocardial tissue sections showing microvessels: (**a**) sham-operated group; (**b**) I/R group; (**c**) GW9662 group; (**d**) telmisartan group; (**e**) telmisartan–GW9662 group; (**f**) candesartan group (CD34 label, 400×). **B**) Quantitative analyses of microvessel cross-sectional area. *P < 0.05 compared with sham-operated; ^#^*P* < 0.05 compared with I/R.

Vascular endothelial cells were visualized by labeling with CD34. The cross-sectional area of microvessels were lower all the treatment groups compared with the sham-operated (80.10 ± 8.47 ***μ***m^2^) group. This decrease in microvessel cross-sectional area was more significant in the I/R (37.17 ± 12.86 ***μ***m^2^) and GW9662 (36.17 ± 4.45 ***μ***m^2^) groups than in the telmisartan (67.19 ± 5.72 ***μ***m^2^), telmisartan–GW9662 (51.49 ± 6.15 ***μ***m^2^), and candesartan (53.97 ± 6.46 ***μ***m^2^) groups. The microvessel cross-sectional area of the telmisartan group was significantly higher than that of the telmisartan–GW9662 and candesartan groups (Figure 3).

### Assessment of myocardial capillary ultrastructure by transmission electron microscopy

**Figure 4:**
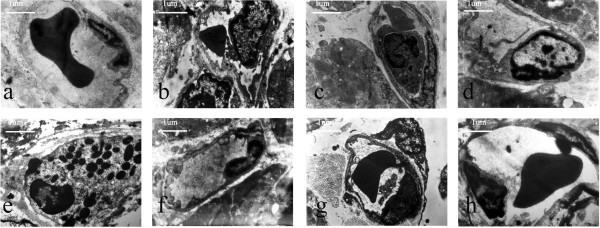
**Photomicrographs of the ultrastructural changes in rabbit capillaries. (a**) sham-operated group, capillary basement membrane was moderate in thickness, and the capillary contained an erythrocyte (7700×); (**b**) I/R group, endothelial cell swelling led to stenosis of the capillary lumen (7700×); (**c**) I/R group, one leukocyte plugged a single capillary (7700×); (**d**) GW9662 group, endothelial cell swelling led to stenosis of the capillary lumen (7700×); (**e**) GW9662 group, one leukocyte plugged a single capillary (7700×); (**f**) telmisartan group, endothelial cell swelling was relieved (7700×); (**g**) telmisartan–GW9662 group, endothelial cell swelling was relieved (7700×); (**h**) candesartan group, endothelial cell swelling was relieved (7700×).

In the sham-operated group, ultramicroscopic examination revealed that the capillaries consisted of endothelial cells. The capillary basement membrane was moderate in thickness, and capillaries contained erythrocytes. In the I/R and GW9662 groups, endothelial cell swelling led to stenosis of the capillary lumen and nearly complete obstruction of the lumen. Close attachment between the leukocyte and the endothelium was observed. The phenomenon of one leukocyte plugged a single capillary was found in the I/R and GW9662 groups. In the telmisartan, telmisartan–GW9662, and candesartan groups, endothelial cell swelling was relieved and stenosis of the capillary lumen was attenuated, while the phenomenon of one leukocyte plugging a single capillary was not found (Figure 4).

### Measurement of myocardial ICAM-1 and VCAM-1 content by ELISA

The myocardial ICAM-1 content of the treatment groups were: sham-operated, 1.36 ± 0.30 ng/mg; I/R, 3.30 ± 0.34 ng/mg; GW9662, 3.19 ± 0.40 ng/mg; telmisartan, 2.06 ± 0.17 ng/mg; telmisartan–GW9662, 2.78 ± 0.20 ng/mg; and candesartan 2.54 ± 0.32 ng/mg. The myocardial VCAM-1 content was: sham-operated, 2.33 ± 0.29 ng/mg; I/R, 3.93 ± 0.45 ng/mg; GW9662, 4.02 ± 0.30 ng/mg; telmisartan, 3.16 ± 0.30 ng/mg; telmisartan–GW9662, 3.54 ± 0.37 ng/mg; and candesartan, 3.38 ± 0.29 ng/mg. The myocardial ICAM-1 and VCAM-1 was significantly higher in all the treatment groups compared with that of the sham-operated group (*P* < 0.05, all). The increase in cardiac ICAM-1 and VCAM-1 content was more significant in the I/R and GW9662 groups than in the telmisartan, telmisartan–GW9662, and candesartan groups (*P* < 0.05, all). Cardiac ICAM-1 and VCAM-1 in the telmisartan group was significantly lower than that of the telmisartan–GW9662 and candesartan groups (*P* < 0.05, all).

### Determination of myocardial NF-***κ***B and PPARG protein expression by Western blot analyses

**Figure 5:**
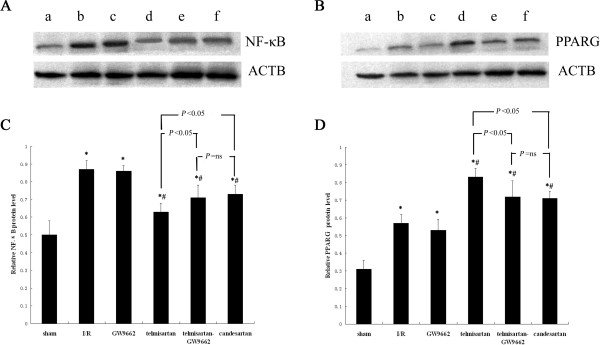
**Determination of myocardial NF-*κ*B and PPARG protein expression by Western blot analyses. A**) Relative expression levels of activated NF-*κ*B protein were determined using Western blot; the IA values were normalized to ACTB expression levels. Lanes **a**, **b**, **c**, **d**, **e**, and **f** in the representative gel show NF-*κ*B expressions in the sham-operated, I/R, GW9662, telmisartan, telmisartan–GW9662, and candesartan groups, respectively. **B**) Relative expression levels of activated PPARG protein were determined using Western blot; the IA values were normalized to ACTB expression. Lanes **a**, **b**, **c**, **d**, **e**, and **f** in the representative gel show PPARG expression in the sham-operated, I/R, GW9662, telmisartan, telmisartan–GW9662, and candesartan groups, respectively. **C**) Quantitative analyses of the expression activated NF-*κ*B protein. *P < 0.05 versus sham-operated group; ^#^*P* < 0.05 versus I/R group. **D**) Quantitative analyses of the expression activated PPARG protein. *P < 0.05 compared with sham-operated; ^#^*P* < 0.05 compared with I/R.

Western blot analyses revealed that NF-***κ***B protein expression observed in all treatment groups was higher compared with that of the sham-operated (0.50 ± 0.08) group. The increase in NF-***κ***B protein expression was more significant in the I/R (0.87 ± 0.05) and GW9662 (0.86 ± 0.03) groups than in the telmisartan (0.63 ± 0.05), telmisartan–GW9662 (0.71 ± 0.07), and candesartan (0.73 ± 0.05) groups. NF-***κ***B protein expression in the telmisartan group was significantly lower than that of the telmisartan–GW9662 and candesartan groups. These results suggest that in the telmisartan (0.83 ± 0.05), telmisartan–GW9662 (0.72 ± 0.09), and candesartan (0.71 ± 0.04) groups, but not the GW9662 (0.53 ± 0.06) group, PPARG protein expression was significantly higher compared with the I/R (0.57 ± 0.05) group (*P* < 0.05, all). PPARG was significantly higher in the telmisartan group compared with the telmisartan-GW9662 and candesartan groups (Figure 5).

## Discussion

The present study showed microvascular dysfunction in the I/R and GW9662 groups, in which microvascular spasm and intracellular/interstitial edema induced a decrease in microvessel cross-sectional area, while neutrophils were adherent and plugged the venules and occasionally the arterioles, with endothelial cell swelling leading to stenosis of the capillary lumen. Furthermore, the myocardial ICAM-1 and VCAM-1 content and NF-***κ***B protein expression were significantly increased during myocardial I/R. Our study also showed that treatment with telmisartan and candesartan exerted protective effects against microvascular dysfunction during myocardial I/R by reducing neutrophil aggregation and adherence, increasing microvessel cross-sectional area, preventing vascular endothelial cells from swelling, and reducing intracellular/interstitial edema. Furthermore, by treatment with telmisartan and candesartan inflammatory marker expression (ICAM-1, VCAM-1 and NF-***κ***B) during myocardial I/R was significantly decreased. In addition, protective effects were more evident in the telmisartan group than in either the candesartan or telmisartan–GW9662 [15]. It is especially important that telmisartan significantly increased PPARG protein expression compared with all other groups.

Recently, the renin–angiotensin system has been shown to have an important role in I/R -induced myocardial injury, confirmed by both indirect and direct studies [27,28]. We found in the present study that angiotensin-II concentrations in left ventricular tissue increased subsequent to myocardial I/R. Activation of NADPH oxidase, stimulated by angiotensin-II, generates reactive oxygen species, which in turn may act as signal transduction messengers for NF-***κ***B. Numerous genes, including interleukin (IL)-1, IL-6, IL-8, interferon-***γ***, TNF-alpha, ICAM-1 and VCAM-1, are stimulated by activation of NF-***κ***B [29]. The adherence and accumulation of neutrophils is mediated by ICAM-1 and VCAM-1 [30]. ARBs ameliorated myocardial I/R-induced pathological inflammation by blocking the AGTR1. This resulted in downregulation of NF-***κ***B expression, which decreased ICAM-1 and VCAM-1 levels, and thus attenuated leukocyte adhesion in the coronary microvasculature. Cell rolling and migration across the endothelium was reduced, and inhibiting inflammatory cell infiltration into the myocardium. The key product of the renin–angiotensin system (angiotensin II) leads to positive inotropism, coronary vasoconstriction, and microvascular spasm by increasing intracellular calcium levels of myocytes and smooth muscle cells [31,32]. Inflammation mediated by neutrophils and microvascular spasm may increase capillary permeability, thus inducing intracellular/interstitial edema formation during myocardial I/R [33]. Both microvascular spasm and microvascular bed compression due to tissue edema [34] may result in a decrease in microvessel cross-sectional area. Changes in total cross sectional vascular area may contribute to the no-reflow phenomenon [34]. ARBs attenuate myocardial I/R -induced intracellular/interstitial edema and microvascular spasms by blocking the AGTR1, resulting in an increase in microvessel cross-sectional area. PPARs are a family of at least three nuclear receptors (***α***, ***δ***, and ***γ***), which regulate genes involved in lipid metabolism, adipocyte differentiation, and inflammation. PPARG activation regulates activating protein-1, signal transducer and the activation of transcription and NF-***κ***B pathways, thereby further inhibiting the production of proinflammatory mediators and cytokines, attenuating oxidative burst, inhibiting inducible nitric oxide synthase induction, and decreasing inflammation [11]. PPARG activation appears to be a novel mechanism for mediating the beneficial effects of ARBs [15,16], and may be directly stimulated by telmisartan [13]. In the present study, histology of the myocardium revealed that microvascular dysfunction was significantly more amended with telmisartan than with either telmisartan–GW9662 or candesartan. Besides the typical effects of ARBs, telmisartan additionally improved microvascular dysfunction through PPARG-mediated effects. PPARG activation may inhibit NF-***κ***B pathways, thus telmisartan further decreased ICAM-1 and VCAM-1 expression, attenuating leukocyte adhesion and infiltration and intracellular/interstitial edema, and further increasing microvessel cross-sectional area. So, telmisartan additionally improved microvascular dysfunction than candesartan, an ARB without PPARG activity. Moreover, telmisartan–GW9662 significantly decreased telmisartan-induced protective effects on microvascular dysfunction by antagonizing PPARG activation.

Recent studies have found that myocardial I/R injury was prevented by ARB in an animal model [35,36]. Furthermore, Hu T et al. [37] reported that chronic pre-treatment of ARB was associated with a reduction in the no-reflow phenomenon in patients with reperfused acute myocardial infarction (AMI) and could preserve microvascular integrity following AMI independent of blood pressure lowering. Therefore, we chose this experimental design to demonstrate that telmisartan has protective effects, in addition to its role as an ARB, against microvascular dysfunction during myocardial I/R via the PPARG pathway. This may be more effective than other ARBs. Moreover, the findings implied that telmisartan may be a more effective therapy than other ARBs for reducing the no-reflow phenomenon in patients with reperfused AMI. However, some limitations of our study deserve consideration. First, we found that telmisartan-GW9662 and candesartan treatment significantly increased PPARG protein expression compared with the I/R group. Jugdutt et al. [36] reported that upregulation of angiotensin type 2 receptor (AGTR2) provides beneficial effects during AGTR1 blockade in a rat model of myocardial I/R. AGTR2 activation may also augment PPARG-mediated effects by enhancing binding of nuclear PPARG coactivators and producing additional endogenous PPARG ligands [38]. This could explain why treatments with telmisartan-GW9662 or candesartan significantly increased PPARG protein expression compared with the I/R group. However, we could not identify which AGTR2 mediated the activation of PPARG-improved microvascular dysfunction in the present study. Further studies will be performed on this important question. Second, our study focused on histology and inflammatory markers and did not include an investigation of blood flow. Measurement of regional myocardial blood flow would be suitable [39]. Therefore, determination of regional myocardial blood flow will be used in our future studies. Third, telmisartan is the most powerful stimulator of PPARG activity among the ARBs, and candesartan is an ARB without PPARG activity [15]. So we chose the two drugs for this experimental design. Moreover,we used equal doses of telmisartan and candesartan in our study, this referred to the research of Ikejima et al. [15]. However, 5 mg/kg/d candesartan may be more powerful than 5 mg/kg/d telmisartan [40]. It is not clear how such differences may affect the comparability of our results. Therefore, administration of different doses of two drugs will be executed in our future studies.

## Conclusion

Taken together, our study suggests that telmisartan has protective effects against microvascular dysfunction during myocardial I/R, in part through PPARG-mediated effects.

## Abbreviations

ACTB: ***β***-actin; AGTR1: Angiotensin type 1 receptor; AGTR2: Angiotensin type 2 receptor; AMI: Acute myocardial infarction; ANOVA: Analysis of variance; ARB: Angiotensin receptor blocker; CD: Cluster of differentiation; ELISA: Enzyme-linked immunosorbant assay; IA: Integrated absorbance; ICAM-1: Intercellular adhesion molecule-1; IL: Interleukin; I/R: Ischemia/reperfusion; LAD: Left anterior descending coronary artery; NF-κB: Nuclear factor kappa-light-chain-enhancer of activated B cells; PMNs: Polymorphonuclear neutrophils; PPARG: Peroxisome proliferator-activated receptor gamma; VCAM-1: Vascular cell adhesion molecule-1.

## Competing interests

The authors declare that they have no competing interests.

## Authors’ contributions

Z XC performed data analyses and wrote the manuscript; Z XC and W H conducted the model building and data collection; L XS have give final approval of the version to be published. All authors read and approved the final manuscript.

## Pre-publication history

The pre-publication history for this paper can be accessed here:

http://www.biomedcentral.com/1471-2261/13/39/prepub
